# Prevalence of medical emergency events in primary dental care within the UK

**DOI:** 10.1038/s41415-023-6444-y

**Published:** 2023-11-10

**Authors:** Melissa Sin, David Edwards, Charlotte Currie, Ian Corbett

**Affiliations:** School of Dental Sciences, Faculty of Medical Sciences, Newcastle University, UK

## Abstract

**Introduction** Dental professionals have an ethical and legal obligation to diagnose and manage medical emergencies which may occur in primary dental care.

**Aims and objectives** To investigate the prevalence of medical emergencies in UK primary dental care and explore the medical emergency training needs of UK primary dental care professionals.

**Design and setting** Cross-sectional survey, targeting dentists, dental hygienists and dental therapists across the UK working within primary dental care.

**Materials and methods** An online survey was distributed via dental society mailing lists, social media groups and a dental conference. The questionnaire consisted of participants demographics and training in, prevalence of and confidence surrounding medical emergencies.

**Results** Respondents (n = 400) were dentists (55.8%), dental hygienists/therapists (38.8%) and specialists (5.5%). Most participants received basic life support training (62%) in 2019, before COVID-19. The most common medical emergencies were syncope, non-specific collapse and hypoglycaemia, being encountered every 1.59, 1.64 and 8.26 years, respectively. The preferred method for medical emergency training was practical based, with more training in administering emergency drugs and equipment identified as a training need.

**Conclusion ** Dental professionals will encounter medical emergencies in primary dental care every 1-2 years and are therefore not uncommon.

**Supplementary Information:**

Zusatzmaterial online: Zu diesem Beitrag sind unter 10.1038/s41415-023-6444-y für autorisierte Leser zusätzliche Dateien abrufbar.

## Key points

- Medical emergencies in primary dental care are encountered by practitioners every 1-2 years.
- It is increasingly important to maintain and develop training in medical emergencies due to current lack in confidence in managing some medical emergencies.
- There is a need for more hands-on training in medical emergencies.

**Supplementary Information:**

Zusatzmaterial online: Zu diesem Beitrag sind unter 10.1038/s41415-023-6444-y für autorisierte Leser zusätzliche Dateien abrufbar.

## Introduction

It is estimated that at least three out of four dentists will encounter a medical emergency in their professional career.^1^ Although there have been studies on medical emergency prevalence outside the UK, data in UK practice are limited and over 20 years out of date.^1^^,^^2^^,^^3^ Furthermore, practitioners' attitudes and experience of medical emergency training remains unexplored.

Diseases which could contribute to medical emergencies encountered by the dental practitioner are increasing in prevalence in the UK. Approximately 7.6 million people suffer with cardiovascular disease, with this number expected to rise due to ageing and growing population.^4^ Similarly, people living with diabetes are expected to increase, affecting one in ten people by 2040.^5^ Polypharmacy is also becoming more common, driven both by the aging population and prevalence of co-morbidities. These, in combination with several other factors, lead to an increased risk of medical complications in dental practice, which the dental care team need to be proficient to manage.^6^

According to the literature outside of the UK, the most common medical emergencies in dental practice include vasovagal syncope, acute angina, seizures and hypoglycaemia.^7^ A British cross-sectional questionnaire from 1999 found the most common medical emergencies to be vasovagal syncope, hypoglycaemia and angina, with dentists seeing an average of 1.9, 0.17 and 0.17 cases annually.^3^

The General Dental Council (GDC) identifies managing medical emergencies as an essential skill that requires up-to-date evidence of capability.^8^ The GDC recommend that dentists maintain their competence by carrying out at least two hours of medical emergency-related continuing professional development (CPD) annually.^9^

Prior to 2013, dental hygienists and dental therapists were required to work under a dentist's prescription - the GDC has now removed the barrier to direct access to these dental professionals for patients. Direct access does not alter the requirement for all registrants to be trained in medical emergencies but may lead to members of the team taking on new roles within a medical emergency.^10^ Within the GDC standards, it is also stated that registrants must be appropriately supported when treating patients, including dental hygienists.^11^ However, responsibility for risk assessment of lone working lies with the individual hygienist; therefore, there is no current consensus for employers that a dental hygienist must work with or without a dental nurse. The GDC states that all registrants must be trained in dealing with medical emergencies, which further emphasises the need for dental therapists and dental hygienists to be confident in managing medical emergencies.^9^

This study therefore aimed to investigate: i) the prevalence of medical emergencies among dentists, dental hygienists and dental therapists working in primary care in the UK; and ii) confidence in their management. This will identify requirements for targeting dental training thereby informing future training needs.

## Materials and methods

The present study was a cross-sectional survey using an online questionnaire distributed through Online Surveys (JISC).^12^ The population of interest was general dental practitioners, dental specialists, dental hygienists and dental therapists across the UK. Ethical approval was obtained from the Newcastle University Ethics Committee before commencement of the study (13543/2020).

Eligibility criteria included all dentists, dental therapists or dental hygienists working within primary dental care in the UK. Recruitment for dentists, dental hygienists and dental therapists were undertaken through: the North of England Dentistry Show Dentistry UK, 2022; the British Society of Periodontology social media page; the Northern Regional Council of Local Dental Committee mailing list; the British Society of Dental Hygiene and Therapy mailing list; the Northern Dental Practice Research Network mailing list; and dental social media groups via Facebook, Instagram and Twitter.^13^^,^^14^ The initial social media posts were sent in September 2021. A reminder was sent two weeks after the initial invitation and before closure of the questionnaire.

The invitation to participate included a participant information sheet outlining the nature of the research and a link to the online questionnaire (which included participant consent). The completion of the questionnaire was accessible to invited participants for a period of five months. Participant-identifiable data were not collected to maintain anonymity; therefore, respondents were not able to withdraw their participation after submission of the questionnaire. An incentive to complete the questionnaire was offered in the form of a competition for a gift voucher.

The components of the survey are outlined in Table 1, with the full questionnaire available in the online Supplementary Information. The questionnaire was piloted with a convenience sample consisting of five dentists working in the UK. Participants were asked to test the usability and content of the questionnaire and provide feedback on the acceptability of the data collection method and the questionnaire. For questions pertaining to the prevalence of medical emergencies, participants were asked about their experience of medical emergencies in 2019, to avoid any potential impact of the COVID-19 pandemic, for example through dental practice closures and reduced patient contact.^15^

**Table 1:** Components of questionnaire

Component	Format
Introduction	Information about the study Screening questions Participation information sheet and consent for participation
Demographics	Sex Year of primary dental qualification graduation Work setting Area code Job title
Training in medical emergencies	Type of training received Pattern of training
Prevalence of medical emergencies	Number of medical emergencies experienced in career and 2019
Confidence surrounding medical emergencies	Diagnosing medical emergencies Managing medical emergencies Carrying out emergency skills Interpreting vital signs as abnormal
Training needs in medical emergencies	Preferred method of learning Personal training needs

Data were exported to Microsoft Excel (v15.39, DC, USA) as a CSV file and cleaned before analysis with descriptive statistics using SPSS (Version 27.0. Armonk, NY, IBM Corp).

## Results

In total, 403 participants were recruited. Three participants were excluded who did not meet the eligibility criteria, leaving 400 included for analysis. These participants were excluded as they were dental nurses. A response rate cannot be calculated due to the open distribution method used. The majority of participants (265/400; 71.3%) identified as female (Table 2). Less than half of participants worked mainly within NHS general dental practice (182/400; 45.5%), with 54.5% of participants working mainly within private general dental practice. Most respondents were dentists (222/500; 55.8%), 38.8% were dental hygienists and/or therapists (155/400) and 5.5% of respondents were dental specialists (22/400).

**Table 2:** Participant demographics

**Individual level variables**	**N/400**	**Valid percent**
**Sex**		
Male	112	28.0
Female	285	71.3
Missing	3	0.7
**Years since qualification**		
0-2 years	25	6.3
3-5 years	28	7.0
6-10 years	52	13.0
11-19 years	87	21.8
20+ years	208	52.0
**Work setting**		
NHS GDP	182	45.5
Private GDP	232	54.5
CDS	42	10.5
NHS specialist practice	13	3.3
Private specialist practice	11	2.8
Out of hours (emergency) dental service	9	2.3
Other	19	4.8
Worked in primary dental care specifically during 2019	347	86.6
**Job role**		
Dental hygienists and dental therapists	155	38.8
Dentist	223	55.8
Dental specialist	22	5.5
Key: GDP = general dental practitioner CDS = community dental services		

### Training in medical emergencies

**Fig. 1:**
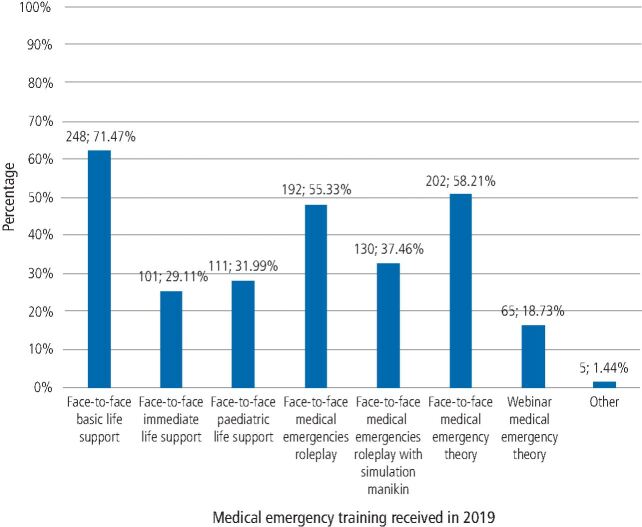
Medical emergency training received by participants in 2019

The majority of participants (347/400; 86.8%) worked within primary dental care in 2019. Medical emergency training received by participants in 2019 included face-to-face basic life support (248/347; 62%), face-to-face theoretical medical emergency training (202/347; 50.5%) and face-to-face medical emergency roleplay (192/347; 48%). Only 32% (130/347) received training involving a simulation manikin (Fig. 1).

Most participants reported undertaking medical emergency training every year (356/399; 89.2%). A small number reported training every six months (27/399; 6.8%), every three months (7/399; 1.8%) and every two years (9/399; 2.3%) (missing data: n = 1). About half of participants (219/400; 49.8%) stated that they did not run or weren't sure if they ran medical emergency scenarios in their practice, with 181/400 not running medical emergency scenarios in their practice.

### Prevalence of medical emergencies

Participants were asked about the prevalence of certain medical emergencies within a specified 12-month period (Table 3). The most common medical emergencies were syncope, non-specific collapse and hypoglycaemia, which dental care professionals would encounter every 1.59, 1.64 and 8.26 years, respectively.

**Table 3:** Prevalence of medical emergencies in 2019

	N		Mean per year	Frequency of occurrence, practice years
	Valid	Missing		
Syncope	347	53	0.6272	1.59
Non-specific collapse	347	53	0.6092	1.64
Hypoglycaemia	347	53	0.1210	8.26
Seizure	347	53	0.0951	10.56
Hyperventilation	347	53	0.0576	17.35
Asthma	347	53	0.0490	20.41
Acute coronary syndrome	347	53	0.0317	31.56
Angina	347	53	0.0288	34.70
Anaphylaxis	347	53	0.0086	115.67
Choking	347	53	0.0058	173.50
Adrenal crisis	347	53	0.0029	347.00
Cardiac arrest	347	53	0.0029	347.00

### Confidence regarding medical emergencies

**Fig. 2:**
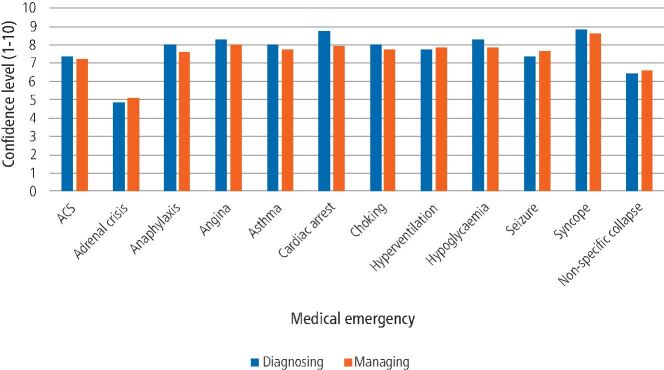
Confidence levels in diagnosing and managing specific medical emergency events (ACS = acute coronary syndrome)

Participants were asked about confidence in diagnosing and managing various medical emergency events (Fig. 2). Among participants, confidence levels for diagnosing and managing medical emergencies was highest for syncope (8.77 and 8.61, respectively) and lowest for adrenal crisis (4.81 and 5.06, respectively).

**Fig. 3:**
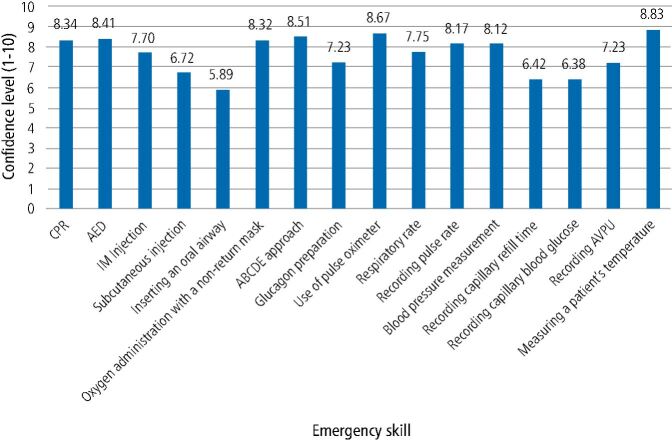
Confidence levels in carrying out emergency skills (CPR = cardiopulmonary resuscitation; AED = automated external defibrillator; ABCDE = airway, breathing, circulation, disability, exposure; AVPU = alert, verbal, pain, unresponsive)

Participants were asked about confidence levels carrying out emergency skills related to the management of medical emergencies (Fig. 3). Among participants, confidence levels were highest for measuring a patient's temperature (8.83) and lowest for inserting an oral airway (5.89).

**Fig. 4:**
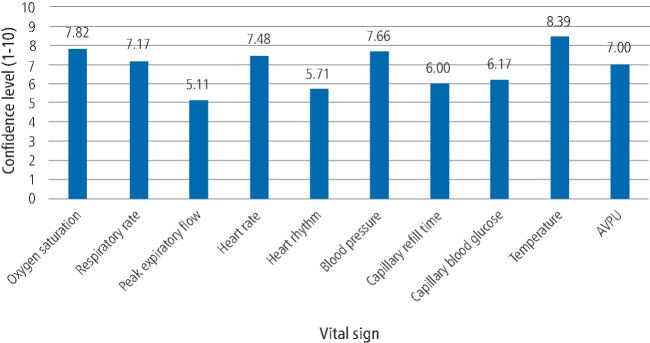
Confidence levels in interpreting the following vital signs as abnormal (AVPU = alert, verbal, pain, unresponsive)

Participants were asked about confidence in interpreting various vital signs as abnormal (Fig. 4). Among participants, confidence levels were highest for interpreting temperature as abnormal (8.389) and lowest for interpreting peak expiratory flow (PEF) reading as abnormal (5.11).

### Training needs in medical emergencies

The majority of participants selected practical-based with interaction, roleplay and/or simulation (372/400; 93%) as a medical emergency training modality. In-person, lecture-based learning was selected by 38% (152/400), interactive webinars by 13.8% and non-interactive webinars by 3.8% (15/400).

The GDC recommends two hours of medical emergency training annually, which 62.5% (250/400) of participants thought was about right and 35.5% (142/400) thought was too little. A small minority (2/400; 0.5%) thought that this was too much and 1.5% (6/400) were not sure.

When asked about personal training needs, 69.2% (258/399) identified needing training in administering emergency drugs and use of emergency equipment. The majority also stated dental team training in managing medical emergencies (260/400; 65.2%) and medical emergencies hands-on/scenario-based training (258/399; 64.7%) were required.

## Discussion

This study is one of the first to investigate dental professionals' attitudes and experiences of medical emergencies training within the UK. Furthermore, previous research into the prevalence of medical emergency events among British dental professionals is limited, being dated and focusing solely on dentists.^3^ Since the introduction of 'direct access' in 2013, a dental professional other than a dentist may be a first responder to a medical emergency; therefore, the present study included dental hygienists and dental therapists.^10^ Generally, a positive attitude towards medical emergencies was found among dental professionals with regards to diagnosis, management, carrying out emergency skills and interpreting vital signs. This reflects similar studies on dentists and dental students within the UK^3^^,^^16^ and may be in part that medical emergency training is compulsory within the UK undergraduate curriculum and is a highly recommended CPD topic by the GDC for all dental professionals.^9^^,^^17^

As of December 2021, 51.5% of dentists on the GDC register were female and 48.5% were male. With regards to dental care professionals (3.5% were dental therapists; 7% were dental hygienists), 92.8% were female.^18^ The participants within the present sample are therefore broadly representative of the UK dental workforce. Furthermore, the results showed a near even split between work within NHS and private dental services, which is also representative; 24,272 dentists performed NHS activity in 2021 and 43,130 dentists were on the UK dental register for 2023.^19^^,^^20^

### Prevalence of medical emergencies

The most common medical emergencies encountered were syncope, hypoglycaemia and non-specific collapse. This aligns with other studies, with the most common medical emergencies being syncope, hypoglycaemia and angina.^3^^,^^19^ This contrasts with the findings of Atherton et al., who found that the most common medical emergency over 20 years ago was seizure (2.8 and 4.0 episodes in 40 years of working careers in England and Wales, and Scotland, respectively).^21^

International studies also aligned with the findings of the present study, with syncope and hypoglycaemia being encountered most frequently. In a 2013 study, less than half of Belgian dentists (237/548; 43.6%) had experienced a medical emergency event in their career to date, with 34.3% reporting vasovagal syncope, 16.1% reporting seizure and 8.4% reporting hypoglycaemia/hyperglycaemia.^22^ A Croatian study found 86.3% of dentists had encountered syncope in a patient^23^ and in another study among French final year dental students, the most common medical emergency experienced was also vasovagal syncope (59.5% of all emergency events recalled).^24^

### Confidence surrounding medical emergencies

Confidence levels for diagnosing and managing medical emergencies were highest for syncope and lowest for adrenal crisis. Participants were confident in measuring a patient's temperature and interpreting this as abnormal but confidence levels were low for inserting an oral airway and interpreting a PEF reading as abnormal. A previous UK-based study had similar findings, where 50.3% and 96.3% of dentists felt comfortable placing an oral airway and managing a syncope episode, respectively,^3^showing that confidence in these skills hasn't changed over the previous 24 years. Furthermore, findings in the study from 1997 included that confidence levels were also high in managing syncope (93.6%) but lowest for anaphylaxis (38%). This aligns with a similar European study, which found high confidence levels in managing vasovagal syncope in both trained and untrained general dentists in medical emergencies.^23^

In the present study, most participants (89.2%) undertook medical emergency training every year, which in part may indicate why overall confidence levels were high in diagnosing, managing and carrying out emergency skills for medical emergencies. Another European study identified low confidence in managing paediatric medical emergencies, specifically administering adrenaline (52.2%) and administering oxygen (43.2%).^23^ Furthermore, it was reported that the majority of participants in this study had never received basic life support training for paediatric patients at undergraduate (81.3%) or postgraduate level (86.1%) and 92.6% of respondents did not keep adrenaline in their practice.^23^ Dental offices in Slovenia have also been found to be insufficiently equipped for the full spectrum of medical emergencies that may occur.^25^ Although the present study did not look at medical emergencies in the paediatric patient, UK resuscitation standards include training on paediatric manikins^26^ and state that adrenaline must be kept in the dental practice.^27^ Findings from the present study differ, with generally high confidence levels among participants in managing anaphylaxis (7.52) and administering oxygen (8.32), which may reflect the frequency of medical emergency training required among UK dental care professionals as set out by the GDC. However, it's important to note that 2.3% of participants undertake training every two years, which goes against the GDC recommendations of two hours of medical emergency training per year for all registrants.^9^ This may put these registrants at risk medico-legally if a medical emergency was to occur with a negative outcome.

In this study, there was a lack of confidence in diagnosing medical emergencies, such as adrenal crisis, anaphylaxis, seizure and non-specific collapse. This could be as, in part, there are currently no formal guidelines with regards to prevention and management of adrenal crisis in a dental setting. This is currently being explored, with guidance being developed,^28^ but importantly, this study highlights current training needs among dental professionals once this guidance is published. Furthermore, there is a lack of confidence in managing additional medical emergencies, such as acute coronary syndrome and anaphylaxis. Confidence in emergency skills were also lower for recording capillary blood glucose, a simple and effective intervention as part of an ill-patient assessment. This suggests that further improvement to current medical emergencies teaching is required to improve dental professionals' confidence surrounding medical emergencies, with personal learning needs for most participants being training in administering medical emergency drugs.

### Preferences for medical emergency training

The most commonly selected method among participants for learning about medical emergencies was practical based with simulation and/or roleplay (93%). This echoes the findings from various other studies, with Kishimoto *et al*. reporting that 100% of participants found simulation to be clinically useful.^29^ Studies with dental students found similar results, with 95.2% recommending a high fidelity simulator for training in medical emergencies.^23^ A Japanese study used a robot patient simulator, with 78% of dental students eliciting positive views.^30^ Studies suggest that simulation training provides statistically significant improvement in the management of medical emergencies.^31^ Despite the evidence of the preference towards roleplay and simulation when learning about medical emergencies, the present study identified that only 32% received training involving simulation and 48% received training involving roleplay. This identifies the disparity between current training opportunities and training needs for dental professionals. It is therefore recommended that all medical emergency training should involve simulation or roleplay to improve learning experiences, which may in turn improve confidence. Studies have shown that confidence may be proportional to training in medical emergencies, with simulation medical emergency training providing more confidence in medical emergency management than didactic learning.^24^

### Limitations

One limitation of this study was that participants were asked to recall the prevalence of an array of medical emergency events in the year of 2019, to avoid interference of the COVID-19 pandemic on the results, due to the closure of dental practices from 25 March 2020.^15^ As a result, this study relies on a participant recall, introducing potential bias and potentially affecting the internal validity of the results.

A sample size of 400 was achieved in this study which may affect its power. In 1999, a similar study based in the UK achieved 1,000 responses. The results are, however, dated, and focuses solely on dentists, with questions including the provision of general anaesthesia in primary dental care, which was ceased in 2001.^32^ This study is therefore the most up-to-date study investigating the prevalence and confidence surrounding medical emergencies and includes the wider dental team, such as dental hygienists and therapists. A power calculation to compare dental hygienists and dental therapists to dentists with regards to the confidence of managing syncope using the two-sample Satterthwaite t-test (assuming unequal variance) identified a sample size of 948 participants would be required using data in the present sample. Therefore, descriptive statistics were used in the present study. Any future studies investigating medical emergency training in different groups of dental professionals should aim for this number.

## Conclusion

Dental professionals will encounter medical emergencies in primary dental care every 1-2 years and are therefore not uncommon. Confidence surrounding medical emergencies varies dependent on the emergency and skills required. This study highlights how important medical emergency training is, identifies requirements for targeting dental training and therefore informs future training needs surrounding medical emergencies.

### Supplementary Information

Supplementary Information (PDF 87KB)
