# Supplementary material for: Prevalence of medical emergency events in primary dental care within the UK
Source: Br Dent J. 2023 Nov 10;235(9):721–6. doi: 10.1038/s41415-023-6444-y (PMC10635819; doi:10.1038/s41415-023-6444-y)
Supplement: Supplementary file 1 — Supplementary Information (PDF 87KB) [file 41415_2023_6444_MOESM1_ESM.pdf]

Part One – Introduction and consent

Thank you for taking the time to look at this survey.
This survey is aimed only at dentists, dental specialists, dental hygienist and/or therapists working within primary care. This includes those working in general dental practice, specialist practice based within a primary care setting and community dental services. If you are not a dentist, dental specialist, dental hygienist and/or therapist working within a primary care setting within the UK please do not complete the survey but thank you for taking the time to look.

The survey will take approximately 5-10 minutes to complete. At the end of the survey you will have the opportunity to provide your e-mail address to enter a prize draw for a £50 Amazon voucher. E-mail addresses will be stored on a password protected server in Newcastle University and deleted once the prize draw winner is contacted.

Ethical approval for this survey has been granted by the Newcastle University Research Ethics Committee (13543/2020). All responses will be stored confidentially and reported anonymously. Completion of the survey is voluntary and you are free to withdraw at any time by exiting the survey. Any responses given prior to withdrawal will be included in analysis.

Do you consent to participate?

Q1: Before agreeing to participate in this study, please read the participant information sheet [Link to participant information sheet].

- I have read the participant information sheet and consent to participate (continue)
- I do not consent to participate

Skip to: Screening message if Answer to Q1 = I do not consent to participate

Screening questions

Q2: Are you a dentist, dental specialist, dental hygienist and/or therapist working within primary dental care setting? This includes general dental practice, specialist practice based within primary care and community dental services.

- Yes (continue)
- No

Skip to: Screening message if Answer to Q2 = No

Q3: Do you work within the United Kingdom?

- Yes (continue)
- No

Skip to: Screening message if Answer to Q3 = No

Part Two – Dental and practice information

Q4: What best describes your gender?	○ Male<br>○ Female<br>○ Non-binary

	○ Transgender Male<br>○ Transgender Female<br>○ Prefer not to say<br>○ Other – please specify
Q5: What year did you complete your primary dental qualification?	○ Drop down list
Q6: Where do you work? (Please select all that apply)	○ General dental practice - NHS<br>○ General dental practice – private<br>○ Specialist practice – NHS<br>○ Specialist practice – private<br>○ Community dental services<br>○ Out-of-hours services<br>○ Other
Q7: What is the first part of the postcode (outward postcode) of the place where you primarily work?	○ Free text
Q8: What is your job title? Tick all that apply	○ Dentist<br>○ Dentist with special interest (if answered, make Q9 available to answer)<br>○ Dental specialist (if answered, make Q9 available to answer)<br>○ Dental hygienist<br>○ Dental therapist<br>○ Dental hygienist and therapist
Q9: What is your specialism?	○ Dental and Maxillofacial Radiology<br>○ Endodontics<br>○ Oral Medicine<br>○ Oral Surgery<br>○ Orthodontics<br>○ Paediatric Dentistry<br>○ Periodontics<br>○ Prosthodontics<br>○ Restorative Dentistry<br>○ Special Care Dentistry<br>○ None

Q10: Did you work the entirety of 2019 within primary dental care?

- ○ Yes (if answered, make Q11-13 available to answer and skip Q14)
- ○ No (if answered, skip to Q14)

Q11: How many sessions a week did you work on average in 2019? A session equates to a morning or afternoon.

- ○ Drop down list
- ○ Other

Q12: What type of training did you receive in 2019 (i.e. before the COVID-19 pandemic)? (Please tick all that apply)

- ○ Face-to-face - Theoretical medical emergencies training
- ○ Webinar-based - Theoretical medical emergencies training

- Face-to-face - Medical emergencies hands-on/scenario based training with a robotic/computer controlled simulation doll (i.e. Sim Man)
- Face-to-face - Medical emergencies hands-on/scenario based training with a role player
- Face-to-face - Basic Life Support (BLS) training
- Face-to-face - Paediatric Immediate Life Support (PILS) training
- Face-to-face Immediate Life Support (ILS) training
- Other – please specify

Q13: During the calendar year 2019 (i.e. before the COVID-19 pandemic), approximately how many of the following medical emergency events have your patients experienced in 12 month period?

	Number
Acute coronary syndrome (myocardial infarction)	○ Drop down list<br>○ Other
Adrenal crisis	
Anaphylaxis	
Angina	
Asthma	
Cardiac arrest	
Choking	
Hyperventilation	
Hypoglycaemia	
Seizure	
Syncope (faint)	
Unspecific collapse	

Q14: What type of training did you receive prior to the COVID-19 pandemic? (Please tick all that apply)

- Face-to-face - Theoretical medical emergencies training
- Webinar-based - Theoretical medical emergencies training
- Face-to-face - Medical emergencies hands-on/scenario based training with a robotic/computer controlled simulation doll (i.e. Sim Man)
- Face-to-face - Medical emergencies hands-on/scenario based training with a role player
- Face-to-face - Basic Life Support (BLS) training
- Face-to-face - Paediatric Immediate Life Support (PILS) training
- Face-to-face Immediate Life Support (ILS) training
- Other – please specify

Q15: What year did you last receive medical emergency training?

- Drop down list
- Other

Q16: On average, how often do you undertake medical emergency training?

- Every 3 months
- Every 6 months
- Every year
- Every 2 years
- Other – please specify

Q17: Do you run regular medical emergency scenarios in your practice?

- Yes
- No
- I don't know

Part Three – Prevalence of Medical Emergencies

Q18: In total, approximately how many of the following medical emergency events have your patients experienced during your dental career?

	Number
Acute coronary syndrome (myocardial infarction)	○ Drop down list<br>○ Other
Adrenal crisis	○
Anaphylaxis	
Angina	
Asthma	
Cardiac arrest	
Choking	
Hyperventilation	
Hypoglycaemia	
Seizure	
Syncope (faint)	
Unspecific collapse	

Part Four – Confidence in managing Medical Emergencies

Q19: How confident do you feel in diagnosing the following medical emergencies?
1: Not confident at all, 10: Very confident

	Number
Acute coronary syndrome (myocardial infarction)	○ 1-10<br>○ I don't know
Adrenal crisis	
Anaphylaxis	
Asthma	
Cardiac arrest	
Choking	
Hyperventilation	
Hypoglycaemia	
Seizure	
Stable angina	
Syncope (faint)	
Unspecific collapse	

Q20: How confident do you feel managing the following medical emergencies within the primary dental care setting?
1: Not confident at all, 10: Very confident

	Number

Acute coronary syndrome	○ 1-10<br>○ I don’t know
Anaphylaxis	
Asthma	
Cardiac arrest	
Choking	
Hyperventilation	
Hypoglycaemia	
Seizure	
Stable angina	
Syncope	
Unspecific collapse	

Q21: How confident would you feel in carrying out the following emergency skills:
1: Not confident at all, 10: Very confident

	Number
Performing cardiopulmonary resuscitation (basic life support)	○ 1-10<br>○ I don’t know
Using an AED	
Providing an intramuscular injection	
Providing a subcutaneous injection	
Inserting an oral airway	
Administration of oxygen with a non-return mask	
The ABCDE approach	
Drug preparation for glucagon administration	
Use of a pulse oximeter	
Recording a respiratory rate	
Recording a pulse rate	
Recording a blood pressure measurement	
Recording a capillary refill time	
Recording a capillary blood glucose	
Use of AVPU (alert, verbal, pain, unresponsive) assessment	
Recording temperature	

Q22: How confident do you feel in interpreting the following vital signs as abnormal?
1: Not confident, 10: Very confident

Oxygen saturation	

Respiratory rate	
Peak expiratory flow	
Heart rate	
Heart rhythm	
Blood pressure	
Capillary refill time	
Capillary blood glucose	
Temperature	
AVPU	

Part Five – Training needs in medical emergencies

Q23: What is your preferred method for learning how to manage medical emergencies? (please tick all that apply)

- In-person lecture based
- Practical based with interaction, roleplay and/or simulation
- Interactive webinar
- Non-interactive webinar
- Other

Q24: The GDC recommend 2 hours of medical emergency training annually. Do you feel this is:

- Too much
- About right
- Too little
- I don't know

Q25: What do you feel your personal training needs are? (tick all that apply)

- Medical emergencies **theory** training
- Medical emergencies **hands-on/scenario** based training (Sim Man)
- Training in administering emergency drugs and equipment
- Basic life support training
- Immediate life support
- Advanced life support
- Dental team training in managing medical emergencies
- Other
- I don't know

Part Six – Prize draw

Thank you for taking the time to complete this survey. If you have any queries, comments or concerns about this survey please contact [research team e-mail].

If you would like to be entered into the prize draw for a chance to a £50 Amazon voucher please provide your email address here [hyperlink to prize draw survey]. Your email address will be stored separately to your survey response and will only be stored until the prize draw. Your email address will not be shared with anyone else and will not be used for any purpose other than the prize draw.

If you would like to know more about medical emergencies, links to some useful resources are provided below:

- https://bnf.nice.org.uk/
- https://www.resus.org.uk/
- https://www.gdc-uk.org/information-standards-guidance/standards-and-guidance/gdc-guidance-for-dental-professionals/medical-emergencies

Thank you

Prize draw survey

Q1: If you would like to be entered into the prize draw for a chance to a £50 Amazon voucher please provide your email address below. Your email address will be stored separately to your survey response and will only be stored until the prize draw. Your email address will not be shared with anyone else and will not be used for any purpose other than the prize draw.

Free text

Thank you
